# Advancements and application prospects of three-dimensional models for primary liver cancer: a comprehensive review

**DOI:** 10.3389/fbioe.2023.1343177

**Published:** 2023-12-21

**Authors:** Liuyang Zhu, Chuanliang Cheng, Sen Liu, Long Yang, Pinsheng Han, Tao Cui, Yamin Zhang

**Affiliations:** ^1^ First Central Clinical College of Tianjin Medical University, Tianjin, China; ^2^ Nankai University of Medicine College, Tianjin, China; ^3^ Department of Pharmacology, Shenyang Pharmaceutical University, Shenyang, China; ^4^ Department of Hepatobiliary Surgery, Tianjin First Central Hospital, Tianjin, China; ^5^ National Key Laboratory of Druggability Evaluation and Systematic Translational Medicine, Tianjin Institute of Pharmaceutical Research, Tianjin, China; ^6^ Research Unit for Drug Metabolism, Chinese Academy of Medical Sciences, Beijing, China

**Keywords:** primary liver cancer, three-dimensional models, bioprinting technology, microfluidic technology, patient-derived xenograft

## Abstract

Primary liver cancer (PLC) is one of the most commonly diagnosed cancers worldwide and a leading cause of cancer-related deaths. However, traditional liver cancer models fail to replicate tumor heterogeneity and the tumor microenvironment, limiting the study and personalized treatment of liver cancer. To overcome these limitations, scientists have introduced three-dimensional (3D) culture models as an emerging research tool. These 3D models, utilizing biofabrication technologies such as 3D bioprinting and microfluidics, enable more accurate simulation of the *in vivo* tumor microenvironment, replicating cell morphology, tissue stiffness, and cell-cell interactions. Compared to traditional two-dimensional (2D) models, 3D culture models better mimic tumor heterogeneity, revealing differential sensitivity of tumor cell subpopulations to targeted therapies or immunotherapies. Additionally, these models can be used to assess the efficacy of potential treatments, providing guidance for personalized therapy. 3D liver cancer models hold significant value in tumor biology, understanding the mechanisms of disease progression, and drug screening. Researchers can gain deeper insights into the impact of the tumor microenvironment on tumor cells and their interactions with the surrounding milieu. Furthermore, these models allow for the evaluation of treatment responses, offering more accurate guidance for clinical interventions. In summary, 3D models provide a realistic and reliable tool for advancing PLC research. By simulating tumor heterogeneity and the microenvironment, these models contribute to a better understanding of the disease mechanisms and offer new strategies for personalized treatment. Therefore, 3D models hold promising prospects for future PLC research.

## 1 Introduction

According to data from the World Health Organization, PLC is the sixth most commonly diagnosed cancer worldwide in 2020 and the third leading cause of cancer-related deaths, imposing a significant global healthcare burden (Forner et al., 2018; Rumgay et al., 2022). Curative treatment for PLC can greatly improve patient prognosis, but the majority of patients have already missed the opportunity and can only resort to systemic therapies, such as targeted therapy and immunotherapy, which have been the focus of attention. However, as of now, the objective response rates of the latest targeted therapy and immunotherapy drugs are both below 30% (Llovet et al., 2022). Apart from host and treatment factors, tumor factors are the main culprits limiting patient benefits, including tumor gene mutations, genomic alterations, expression status of immune checkpoints, tumor heterogeneity, and the tumor microenvironment. The maturation of Next-generation sequencing and Fluorescence *in situ* hybridization technologies has made it economically feasible to understand the genomic status of patients. However, the tumor microenvironment and tumor heterogeneity remain significant challenges in the field of cancer research. Tumor heterogeneity refers to the presence of different cell populations within a single tumor, leading to high variability in terms of genetic, phenotypic, and behavioral characteristics (Tabrizian et al., 2015; Kudo et al., 2018). This diversity stems from factors such as gene mutations, epigenetic modifications, and interactions with the tumor microenvironment, resulting in drug resistance, tumor recurrence, and metastasis. The tumor microenvironment consists of various cellular and non-cellular components that surround and interact with cancer cells (Anderson and Simon, 2020). These components play a crucial role in tumor growth, progression, immune evasion, and response to treatment. Tumor heterogeneity refers to the possibility that multiple subpopulations of cancer cells within a single tumor may exhibit different sensitivities to targeted or immunotherapies.

The lack of preclinical models that can reproduce genetic heterogeneity and the tumor microenvironment has greatly limited the in-depth study of the molecular mechanisms of PLC and the development of personalized therapies. As commonly used *in vitro* models for PLC, traditional 2D cell lines, genetically engineered mouse models, and chemically-induced mouse models are still widely used in preclinical research and play an irreplaceable role in exploring the pathogenesis of PLC and drug responses (Sharma et al., 2010; De Minicis et al., 2013; Shamir and Ewald, 2014).

The limitations of 2D cell lines cultures are evident and can be summarized as follows: Firstly, there is a lack of tissue structure. The 2D flattened structure is unable to simulate the cell-cell and cell-extracellular matrix interactions that occur in the original tumor microenvironment. Secondly, there is low representativeness. Despite the availability of numerous liver cancer cell lines for research, they still struggle to represent the heterogeneity observed in liver cancer patients. Thirdly, there are changes in gene expression and phenotype. Due to the artificial environment of cell culture, significant differences may exist between cells cultured in 2D and the key cellular characteristics, such as metabolism, differentiation status, and cell signaling pathways, observed *in vivo*. These differences can lead to variations in treatment responses. Lastly, there is a weak clinical translational capability. 2D culture models may not effectively reflect the response to potential therapies *in vivo* due to differences in drug penetration, cellular responses, and microenvironmental factors.

Genetically engineered mouse (GEM) models of liver cancer are time-consuming and labor-intensive to design and produce. Additionally, genetic manipulations can inadvertently affect multiple pathways, making the interpretation of data highly complex. Chemically-induced mouse models of PLC cannot accurately represent the genetic and molecular characteristics of specific human liver cancers, nor can they fully capture the complex interactions between tumor cells and the surrounding microenvironment in PLC. Moreover, the safety concerns associated with toxins raise ethical issues in animal research (De Minicis et al., 2013).

**FIGURE 1:**
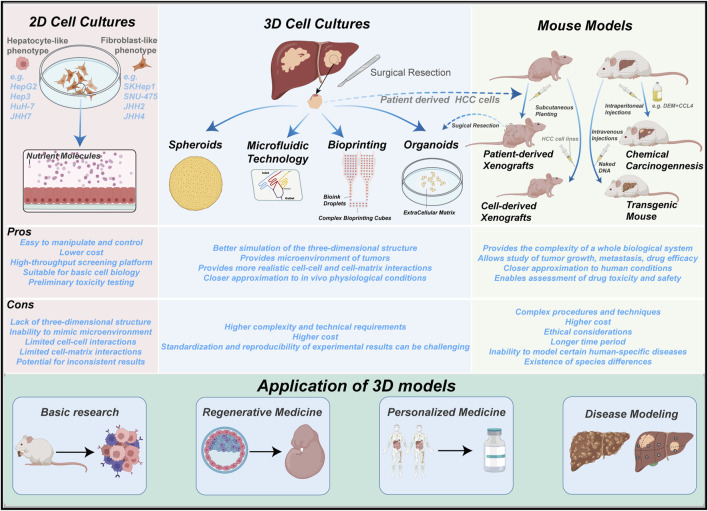
Summary of the main experimental models in PLC. 2D cell cultures, mouse models and 3D cultures. 2D cell cultures are the simplest to operate, yet observations may deviate from the true *in vivo* biological processes. Mouse models better recapitulate the *in vivo* environment, but further genetic manipulations are not possible after formation of tumors. 3D cultures emerge as a superior model which incorporates their strengths.

To overcome the limitations of traditional liver cancer models, various 3D culture models have emerged, taking advantage of the advancements in cell culture and microfabrication technologies. Particularly, emerging biomanufacturing techniques such as 3D bioprinting, microfluidics, and organ-on-a-chip models have greatly facilitated the development of novel liver cancer models. Compared to traditional models, 3D culture models can induce different cell morphologies and physiological characteristics, more accurately mimicking the tumor microenvironment *in vivo* and reproducing cell polarity, morphology, tissue stiffness, and cell-cell interactions. Figure 1 provides an overview of the main existing models for studying PLC.

This review summarizes and discusses the latest advancements in 3D models for PLC and their application value in tumor biology, pathogenesis research, and drug screening.

## 2 The establishment of 3D models of PLC

### 2.1 Traditional 3D cell culture techniques

Traditional 3D cell culture involves the use of special cultivation methods or the construction of multicellular culture models with spatial 3D structures based on matrices, scaffolds, and other special materials. It can generally be divided into two types: scaffold-based or scaffold-free 3D culture models.

#### 2.1.1 Scaffold-based 3D models

The scaffold-based 3D culture technique utilizes artificial support materials that mimic the extracellular matrix, providing a fixed point for the adhesion and growth of liver cancer cells. Cells can then proliferate and migrate within the scaffold, acquiring typical characteristics of *in vivo* tumors. These scaffolds are primarily designed using natural materials such as collagen, hyaluronic acid, and gelatin, or synthetic polymers such as polycaprolactone, polyethylene glycol, and polylactic acid, in order to generate a cell-free matrix or hydrogel (Drury and Mooney, 2003). Natural materials exhibit better biocompatibility and lower toxicity, while synthetic polymers offer better manipulability and multifunctionality. Additionally, 3D porous scaffolds constructed based on biomaterials provide physical structural support for *in vitro* cell culture and *in vivo* tissue regeneration.

Hydrogels, due to their biocompatibility and similarity to the extracellular matrix, are widely used in 3D culture of liver cancer cells. Madhushree et al. utilized a plant-derived natural nanofibrillated cellulose hydrogel for 3D culture of HepG2 liver cancer cell line and found that it promoted cell proliferation and differentiation, showing great potential in drug testing, tissue engineering, and cell therapy (Bhattacharya et al., 2012). Greene et al. encapsulated Huh7 liver cancer cells in a gelatin-based hydrogel, which enhanced cellular metabolic activity (Greene and Lin, 2015). The microstructure and biochemical composition of natural extracellular matrix can be preserved in decellularized matrix, providing a tissue-specific microenvironment for the growth of normal liver cells and liver cancer cells (Peng et al., 2018). Calitz et al. encapsulated liver cancer cells (Huh7), hepatic stellate cells (LX-2), and human umbilical vein endothelial cells (HUVEC) in a mixture of collagen and fibrinogen to construct a 3D liver cancer biomimetic model. Compared to the 2D model, this model exhibited significantly enhanced tumor proliferation, migration, and resistance to chemotherapy drugs (Calitz et al., 2020).

Porous microscaffolds constructed from hydrogels or other biomaterials have also been used to build 3D models of liver cancer, including hydrogel microspheres, hydrogel interlayers, hydrogel porous microspheres, or 3D bioprinted hydrogel structures (Liaw et al., 2018). The porous scaffolds provide a larger contact area for cell adhesion and facilitate cell-cell interactions. Moscato et al. mixed polyvinyl alcohol with gelatin, freeze-dried it repeatedly, and formed porous scaffolds with pore sizes ranging from 30 to 150 μm HepG2 liver cancer cells were cultured in the 3D scaffolds, resulting in the formation of tumor spheroids with the longest cell survival reaching 21 days and the presence of necrotic regions in the center (Moscato et al., 2015). Chen et al. used microfluidic technology to fabricate poly (lactic-co-glycolic acid) (PLGA) porous microspheres with an average diameter of 395 μm and pore size distribution ranging from 10 to 60 μm (Wang et al., 2020). They dynamically cultured HepG2 cells in the porous microspheres and added HUVEC to promote tumor vascularization and interconnection between microspheres. This model allowed the observation of tumor microtissue formation and exhibited significantly higher IC50 values compared to traditional 2D culture conditions, indicating better drug resistance of cells in 3D culture and a closer resemblance to real tumor tissues. Leung et al. cultured liver cancer cell lines PLC/PRF/5 and HepG2 in chitosan-alginate scaffolds to construct 3D liver cancer models (Leung et al., 2010). The results showed increased expression of malignant markers IL-8, bFGF, VEGF, and GPC-3 in the 3D models. Furthermore, compared to 2D or matrix gel culture, the hepatocellular carcinoma (HCC) cells cultured in the 3D scaffolds exhibited significantly enhanced resistance to doxorubicin.

The construction of liver cancer 3D models using novel material scaffolds provides a tumor matrix environment that more closely resembles *in vivo* conditions, facilitating cell growth and proliferation (Nikolova and Chavali, 2019). The porous nature of the scaffolds enables oxygen and nutrient exchange, drug delivery, and clearance of waste molecules. 3D scaffold models are of great significance and have potential applications in studying liver cancer molecular biology, cell interactions within the tumor microenvironment, and anti-cancer drug development and screening.

#### 2.1.2 Scaffold-free 3D models

A scaffold-free 3D model of liver cancer requires special culturing methods to induce cell aggregation and the formation of so-called “tumor spheroids,” where the only extracellular matrix component present is produced by the tumor cells themselves. Currently, commonly used culturing methods include rotating bioreactors, hanging drop cultures, suspension cultures, and the latest development of microfluidic technology. The main purpose of these methods is to promote self-assembly of tumor cells and their adhesion to form spherical structures, thereby maximizing cell-cell interactions.

Cui et al. cultured liver cancer cells and normal liver tissue fragments in a rotating wall vessel bioreactor for 3D rotation mixing, and found that liver cancer cells aggregated around the normal liver tissue and formed spheroids, recapitulating the pathological process of liver cancer invasion and metastasis. This model can serve as a novel *in vitro* model for liver cancer. Teresa et al. used the rotating culture method to construct a 3D spherical model of breast cancer with fibroblasts and endothelial cells, which could be cultured long-term and used to investigate cell-cell interactions and the potential impact of endothelial cells on drug response within the tumor microenvironment (Franchi-Mendes et al., 2021). Tang et al. utilized a rotating wall vessel bioreactor to culture liver cancer cells on a molecular scaffold, constructing a 3D *in vitro* model of liver cancer with high metastatic ability. Tumor morphology and biochemical analysis indicated that the 3D model reflected many clinical pathological features of liver cancer, including cell morphology, tissue ultrastructure, specific gene expression, and cell apoptosis (Tang et al., 2011). Shah et al. used HepG2 cells to construct a 3D liver cancer model through hanging drop culture, aiming to explore the growth characteristics of tumor spheroids and the applicability of gene toxicity testing (Shah et al., 2018). Yip et al. co-cultured liver cancer cells and fibroblasts to form spheroids using the hanging drop method, embedding them in a collagen hydrogel to construct a liver cancer 3D model (Yip and Cho, 2013). Drug resistance was found to be stronger in the 3D model during drug testing, providing a good preclinical model for drug development. Hrout et al. cultured HepG2 liver cancer cells and fibroblasts to form tumor spheroids using ultra-low attachment culture plates (Al Hrout et al., 2022). Compared to traditional 2D models of liver cancer cells alone, this model exhibited significantly increased expression of poor prognostic factors such as tumor proliferation and migration. Additionally, studies have found that using magnetic nanoparticles to provide a magnetic environment for cells enables magnetic suspension culture, preserving cell characteristics during the formation of 3D tissues (Caleffi et al., 2021). Other studies have promoted cell adhesion and the formation of tumor spheroids by adding nanofibers to the cell suspension (Lee et al., 2021).

#### 2.1.3 The limitations of traditional 3D models of PLC

Regardless of whether scaffold technology is used, the construction of a 3D model of PLC through culturing increases the dimensionality of cells and allows for a better representation of the tumor environment *in vivo*. Moreover, the 3D model facilitates cell-cell interactions, and co-culturing with stromal cells such as tumor-associated fibroblasts and endothelial cells enables the exploration of the tumor microenvironment and the mutual influence between liver cancer cells and the stromal cells.

Generally speaking, traditional 3D cell culture methods still have some limitations. For scaffold-based 3D models, although natural ECM-derived matrix materials provide binding sites for cells, tumor cells exhibit similar biological behavior *in vivo*. However, natural materials are usually subject to different species sources, individual differences, separation, and purification methods, resulting in different composition and properties between batches, which will have a great impact on the repeatability of the study (Brancato et al., 2020). For synthetic material scaffolds, although they have advantages in structural controllability, stability, differences between batches and biomechanical control. However, the lack of binding sites such as cells or factors makes them need functional chemical modification. Natural and synthetic scaffolds and matrix materials have their own characteristics, perhaps the combination of the two can further improve the effect of ECM simulation of tumors *in vivo*. For the scaffold-free 3D model, the complex operation limits the uniformity and repeatability of the spheroid. In addition, the traditional 3D cell culture also needs to simplify the culture program to achieve high-throughput screening technology, which is of great significance for individualized therapy and drug screening (Yun et al., 2022).

### 2.2 Microfluidic technology for constructing "organ-on-a-chip"

Microfluidic perfusion culture systems, also known as “organ-on-a-chip,” can be made of plastic, glass, or synthetic polymers and allow for the co-culturing of various cell types, including tumor cells, endothelial cells, and stromal cells. Microfluidic technology ensures continuous perfusion of oxygen and nutrient supply, further enhancing the ability of *in vitro* models to mimic tumor physiology. The diversity of microfluidic designs with different channels allows for precise control of the spatial distribution of different cell types and biochemical gradients.

In complex liver cancer 3D *in vitro* models, a functional vascular system is crucial. Blood vessels can be formed in microfluidic devices by seeding endothelial cells in channels or by utilizing the self-organizing properties of endothelial cells in co-culture with fibroblasts. The latest developments in microfluidic technology have made it an important tool for 3D cell culture and drug testing, particularly in mimicking the *in vivo* tissue microenvironment with high reproducibility (van Duinen et al., 2015). Siming Lu et al. developed a biomimetic 3D liver cancer organ-on-a-chip that simulated the tumor microenvironment by integrating decellularized liver extracellular matrix and GelMA into a microfluidic culture system (Lu et al., 2018). This system contained necessary scaffold proteins and growth factors, promoting the proliferation and growth ability of HepG2 liver cancer cells as well as liver cell functionality. The organ-on-a-chip exhibited a linear dose-dependent drug response to acetaminophen and sorafenib toxicity. Lee et al. constructed a microfluidic 3D model of the tumor microenvironment of HCC with hepatitis B virus (HBV)-positive cells (Lee et al., 2018). This model was used to study the immunosuppressive effects of monocytes on HBV-specific T cell receptor redirected T cells (TCR-T cells) and the role of PD-L1/PD-1 signaling pathway. The results showed that this microfluidic model was more effective than 2D models in predicting the efficacy of TCR-T cell therapy for HCC and could be used to improve current immunotherapy strategies. Zuchowska et al. used a microfluidic system for long-term culture of liver cancer 3D cell spheroids to determine the effects of the chemotherapeutic drug 5-FU on liver cancer cells. The viability of HepG2 spheroids was rapidly analyzed using a chip scaffold microplate reader for fluorescence analysis (Zuchowska et al., 2017).

Microfluidic technology allows for precise control of cell-cell interactions, enabling the co-culture of different types of tumor cells and stromal cells to recreate the tumor microenvironment *in vitro*. Additionally, microfluidic chips provide shear effects similar to physiological microenvironments, offering a new method for drug delivery (Ahn et al., 2017). Currently, liver cancer organ-on-a-chip models provide a novel preclinical model for studying the mechanisms and drug treatments of PLC. They are also valuable for the development, evaluation, and screening of novel anticancer drugs.

The challenges faced by microfluidic models include high cost, complex fabrication processes, and difficulties in handling. Current microfluidic models predominantly use polydimethylsiloxane (PDMS) combined with glass to ensure chip transparency. However, PDMS material itself is costly, and the manufacture and commercialization of microfluidic platforms require substantial expertise and equipment, further increasing the overall cost (Kuo et al., 2023; Strelez et al., 2023). Moreover, the fabrication and commercialization processes of microfluidic models are relatively complex. Despite the availability of plug-and-play microfluidic systems in the market, individuals without a fundamental knowledge of these systems still need to acquire specialized technical skills and operational proficiency, which may discourage some users. Operating microfluidic models demands high levels of dexterity as they utilize small volumes and dimensions, necessitating the avoidance of bubble formation and excessive shear forces to maintain cell viability and behavior. Additionally, due to limited space, operators need specialized training to handle these systems proficiently. Nevertheless, microfluidic models remain promising *in vitro* tools for studying cellular behavior and evaluating drug efficacy, as they enable the use of low volumes of therapeutics and cell samples within the chip, ultimately enhancing cost-effectiveness (Ao et al., 2022).

### 2.3 3D bioprinting

3D bioprinting is an emerging tissue engineering technology that builds on the principles of computer-assisted traditional rapid prototyping in 3D printing. It utilizes bioinks composed of hydrogels, cells, growth factors, and other components to print intricate microstructures of biological materials layer by layer on a culture medium or substrate. By finely controlling the quantity and types of cells, composition of the extracellular matrix, deposition of cell-matrix scaffolds, and dynamic microenvironment, 3D bioprinting can simulate complex tissue structures with biologically active properties to a certain extent. It has become an important technique for constructing complex *in vitro* tissue models. There are various methods of 3D bioprinting, including extrusion-based bioprinting, laser-assisted bioprinting, inkjet-based bioprinting, magnetic bioprinting, coaxial bioprinting, and acoustic bioprinting, which differ in their underlying principles (Xiang et al., 2022; Zhuang et al., 2023).

Extrusion-based bioprinting is the most common technique, which involves the precise deposition of cells using a fluid dispensing system, allowing for the combination of bioinks and cells for bioprinting (Tetsuka and Shin, 2020). Even complex tissues can be printed using multiple channels and different bioinks, showcasing its advantages of cost-effectiveness and high cell viability (Zhuang et al., 2023). Inkjet bioprinting, derived from 2D inkjet printers, utilizes the printing of bioink droplets to precisely control the volume and size of tissue pattern samples, making it efficient and simple. Inkjet bioprinting can be further categorized into on-demand inkjet printing and continuous inkjet printing, depending on the continuity of inkjetting. However, it has some drawbacks. Firstly, the precision of droplet inkjetting is affected by nozzle clogging and cell deposition in the printing chamber. Secondly, inkjet printing requires high electrical conductivity of bioinks, posing challenges in ink selection. Lastly, the cell viability in inkjet printing is not satisfactory. Laser-assisted bioprinting utilizes the principle of laser-induced forward transfer, enabling precise control of virtual deposition of cells and biomaterials with higher resolution. Since it does not involve nozzle processes, cell viability is guaranteed. However, the high cost of equipment limits its development. The selection of hydrogels in bioinks is crucial as they provide the microenvironment for cell proliferation and differentiation (Huang et al., 2017). Therefore, factors such as biocompatibility, flowability, polymer properties, and biodegradability need to be considered in the selection of hydrogels for bioinks (Decante et al., 2021).

Bioprinting technology allows for precise positioning of cells and biomaterials, reproducing the composition and spatial complexity of the tumor microenvironment (Liu et al., 2020). It provides a promising biomanufacturing technique for constructing stable 3D *in vitro* models of liver cancer. Mao used a gelatin-alginate-MatrigelTM composite hydrogel to bioprint 3D *in vitro* models of intrahepatic cholangiocarcinoma using patient-derived cells. The models exhibited significantly higher cell proliferation capacity and levels of tumor markers compared to 2D models, as well as increased resistance to chemotherapy drugs and targeted drugs (Mao et al., 2020). Changcan utilized bioprinting technology to construct 3D models of cholangiocarcinoma and co-cultured tumor cells with tumor-associated endothelial cells, tumor-associated fibroblasts, and tumor-associated macrophages (Li et al., 2022). The 3D bioprinted models demonstrated enhanced proliferation capacity, higher levels of tumor-associated gene expression, and increased drug resistance, with the presence of stromal cells promoting the activity of tumor cells. Xie et al. mixed patient-derived HCC cells with gelatin and alginate, and used 3D bioprinting to establish personalized liver cancer *in vitro* 3D models (Xie et al., 2021). The analysis showed that the models retained the characteristics of the parental HCC, including stable expression of biomarkers and consistent gene expression profiles. The models also accurately displayed the results of anticancer drug screening, providing significant value for personalized treatment of liver cancer patients.

Decellularized and solubilized extracellular matrix (dECM) is a potential biological ink with tissue-specific components. however, the poor gel kinetic properties of ECM limit the accuracy of 3D biological printing. Martina et al. invented a tissue-specific composite biological ink, which is composed of natural polymer alginate and enhances its biological activity with dECM. ECM enhances the survival of primary human progenitor cells during 3D biological printing, supports tissue-specific cell differentiation, and stimulates full-layer vascularization of implants *in vivo* while minimizing allogeneic reactions (De Santis et al., 2021). Min Kyeong et al. developed a novel bioprinting technology for dECM-incorporated hepatocyte spheroids that could enhance both cell-cell and -ECM interactions simultaneously. The dECM materials were uniformly distributed in the bioprinted spheres and the incorporation of dECM significantly improved the liver function of hepatocyte spheres (Kim et al., 2023). In general, dECM biological ink can be used to construct tissue and organ-specific microenvironment, which can determine cell fate and tissue development *in vitro* (Kim et al., 2020).

In order to enhance the biomimicry and complexity of the models, the combination of 3D printing and microfluidic technology has become a hot topic in the field of biomanufacturing and drug screening research. With the rapid development of printing devices and novel bioinks, extrusion-based printing methods have become a feasible choice for constructing organ-on-chip models (Yi et al., 2017). Microfluidic channels can be fabricated by printing bioinks such as hydrogels, followed by printing cell-laden bioink mixtures into the existing channels (Bertassoni et al., 2014; Bhise et al., 2016). This printing method improves the efficiency and reproducibility of constructing drug screening chip models, and it is an important direction for the integration of 3D printing and microfluidic technology.3D printing is used for the preparation of liver cancer cell spheroids, allowing for precise control of cell quantity and spheroid size, while microfluidic chips can provide a more biomimetic *in vitro* microenvironment for the cells (Ouyang et al., 2015). Li et al. integrated the advantages of 3D bioprinting, microfluidics, and hydrogel technology to construct a controllable-sized liver cancer 3D bioprinting-microfluidic model (Li Y. et al., 2019). This model was applied in pharmacological experiments with a novel anti-CD147 monoclonal antibody, Metuzumab, providing important reference value for the construction of complex *in vitro* liver cancer models and antibody drug screening research. Furthermore, 3D bioprinting has promoted the development of tissue and organ regeneration engineering. Previous studies have shown that using bioprinting technology to construct liver tissue models with primary hepatocytes, endothelial cells, and fibroblasts can be applied in liver tissue regeneration, artificial liver, and liver transplantation in the future (Lee et al., 2016; Lei and Wang, 2016).

Indeed, 3D bioprinting of tumor models can provide a more accurate replication of the complex tumor microenvironment and treatment response *in vivo*, offering significant potential for personalized treatment of liver cancer and the development of anticancer drugs (Meng et al., 2019). However, many of the currently established 3D bioprinted liver tumor models still cannot fully replicate the complete physiological characteristics of tumors, including the vascular microenvironment and immune microenvironment. Further improvements are still needed in these areas (Kronemberger et al., 2021).

### 2.4 Liver cancer organoids

Organoids derived from adult stem cells (ASC), embryonic stem cells (ESC), or induced pluripotent stem cells (iPSC) have been generated to study the occurrence and development of liver cancer (Schwank et al., 2013; Bershteyn et al., 2017; Turner et al., 2017; Nguyen et al., 2021). The liver organoid model provides a powerful and genetically flexible platform for cancer research. Previous studies have shown that the construction of liver organoids requires various cytokines and growth factors, including hepatocyte growth factor (HGF), epidermal growth factor (EGF), fibroblast growth factor and R-spondin 1 (Rspo1), TNFα, Wnt agonists, LGR5 ligands, cAMP agonists, and TGF-β inhibitors (de Lau et al., 2011; Broutier et al., 2016; Peng et al., 2018). Currently, organoids have been used to construct models of fatty liver disease (Ouchi et al., 2019), liver cancer (Broutier et al., 2017), drug-induced liver injury (Shinozawa et al., 2021), cholestatic liver injury (Sato et al., 2021) and HBV-related hepatocellular carcinoma (De Crignis et al., 2021).

Liver Cancer Organoids can be established based on healthy liver organoids through chemical treatment or genetic engineering editing, but their primary source is still liver cancer cells (Artegiani et al., 2019; Naruse et al., 2020). Some studies have utilized normal liver tissue to construct organoids (TP53WT), and then used CRISPR technology to knock out TP53 in the liver organoids (TP53KO). The TP53R249S mutant variant was overexpressed in the TP53 knockout organoids using a lentiviral vector. Finally, these three types of organoids were transplanted into mice to establish an ODX model, and tumor progression was observed to investigate the role of TP53 and R249S in the occurrence and development of HCC (Lam et al., 2023).

Patient-derived liver cancer organoids retain key characteristics and genetic mutations of the original tumor and have been widely used in tumor mechanism research and drug screening (Bresnahan et al., 2020). Qiang Gao et al. established a patient-derived liver cancer organoid biobank (LICOB) that comprehensively represents the histological and molecular characteristics of various liver cancer types as determined by multiomics profiling, including genomic, epigenomic, transcriptomic, and proteomic analysis. And the LICOB is a rich resource for investigation of liver cancer biology and pharmacological dependencies and may help enable functional precision medicine (Ji et al., 2023). Ling Li et al. screened 129 anti-tumor drugs using patient-derived liver cancer organoids and found significant differences in the efficacy of clinically used drugs, such as sorafenib and gemcitabine, among patients with PLC (Li L. et al., 2019). However, a small number of non-liver cancer indication drugs, such as pucathromycin and idarubicin, may be effective. Lulu Sun et al. used hiHEP self-assembly to form liver organoids with liver-polarized structures and enhanced liver function (Sun et al., 2019). By introducing different carcinogens to simulate the occurrence of HCC and ICC, this model can be used to explore molecular and cellular changes in early hepatocarcinogenesis and develop new preventive strategies. Broutier et al. successfully constructed tumor organoids derived from eight patients with PLC, including HCC, cholangiocarcinoma (CC), and mixed hepatocellular carcinoma (CHC) (Xian et al., 2022). After long-term culture, these organoids recapitulated the tissue structure, expression profile, genomic landscape, and *in vivo* tumorigenicity of the parental tumors. Additionally, the research team used the PLC organoids as biomarker identification and drug screening models and identified the ERK inhibitor SCH772984 as a potential therapeutic agent for PLC. This study directly demonstrates the utility of organoids in identifying genes and potential new therapeutic targets with prognostic value for liver cancer, thus opening opportunities for advances in drug testing and personalized medicine approaches (Broutier et al., 2017). Broutier believes that PLC-derived organoids are complementary and alternative models to liver cancer patient-derived xenografts (PDX), and the significantly reduced construction time makes them more suitable for large-scale drug testing and tumor individualized therapy. Linfeng Xian et al. established 52 organoids from 153 PLC patients. Compared to PDX models, the establishment of HCC organoids had a higher success rate (29.0% vs 23.7%) and shorter time (13.0 ± 4.7 vs 25.1 ± 5.4 days) (Xian et al., 2022). The organoids and ODX reproduced the histopathological features of PLC, and it was found that targeting the mTOR signaling pathway could overcome acquired sorafenib resistance in liver cancer organoids by inducing phosphorylation of S6 kinase. Nuciforo et al. constructed organoid models using tumor tissue and adjacent non-tumor tissue from different types of liver cancer patients through biopsy. The success rate was related to pathological grading and Ki67. Sorafenib drug response experiments revealed dose-dependent inhibition of HCC organoid growth, demonstrating that PDOs are suitable for preclinical drug development validation (Nuciforo et al., 2018).

There is limited research on the use of liver cancer organoid models in personalized immunotherapy. Wenwen Wang et al. conducted a comparative analysis of 27 constructed liver and bile tumor organoids with the original tumor tissue and confirmed that the organoids retained the genetic characteristics, HLA-class-I phenotype, and neoantigen-related mutation profiles of the parental tumor tissue to a great extent (Wang et al., 2022). By using multi-omics approaches, they predicted and analyzed the neoantigen peptide libraries of liver and bile tumors, established an organoid-based platform for *in vitro* screening of neoantigen peptides, and preliminarily demonstrated the tremendous potential of this platform in evaluating the efficacy of personalized immunotherapy.

Undeniably, organoids have greatly facilitated the development of *in vitro* models for liver cancer and are of significant importance in expanding tumor biobanks, investigating the mechanisms of liver cancer development (Jia et al., 2022), drug response testing, and personalized medicine. Single-cell transcriptome analysis delineates heterogeneity of hepatobiliary tumor organoids and proposes that the collaboration of intratumoral heterogenic subpopulations renders malignant phenotypes and drug resistance (Zhao et al., 2021). However, there are still limitations to organoids (Tuveson and Clevers, 2019). They lack various cells that constitute the tumor microenvironment, including endothelial cells (Lim et al., 2022), immune cells, and fibroblasts (Shiota et al., 2021; Badr-Eldin et al., 2022; Dong et al., 2022). Fibroblasts have been shown to promote tumor growth, metastasis, and chemotherapy resistance (Zhang et al., 2018), while immune cells play a crucial role in the tumor microenvironment (Dijkstra et al., 2018). Introducing different stromal cells may improve the reliability of liver cancer organoids. Moreover, it was observed that healthy contaminating tissue within the samples gave rise to organoids that would quickly outcompete the tumour-derived organoids, so it is necessary to optimize the culture program. Additionally, PLC exhibits inter- and intra-tumoral heterogeneity, and reproducing tumor heterogeneity in liver cancer organoids is one of the challenges that need to be addressed. Furthermore, improving the success rate of organoid construction, reducing the time required for organoid generation, and lowering the cost of organoids are also important challenges (Tuveson and Clevers, 2019).

### 2.5 Patient-derived xenograft (PDX) models

Patient-derived xenograft (PDX) models are established by transplanting patient tumor tissues into immunodeficient mice. The first HCC PDX model was successfully established in 1996, but its development has been slow, and the success rate remains relatively low. As a more reliable *in vivo* tumor model, PDX models retain the tissue structure, gene expression profile, and drug response characteristics of the patient’s primary tumor (Brown et al., 2018; Xu et al., 2021). Subcutaneous transplantation is the most commonly used method for establishing liver cancer PDX models, which allows for more accurate measurement of tumor growth and drug response. Renal subcapsular transplantation further improves tumor engraftment due to its rich blood supply (Gao et al., 2015). In addition, orthotopic transplantation into the liver parenchyma can more faithfully replicate the tumor microenvironment, including interactions with blood vessels, stromal cells, and immune cells, and better simulate tumor metastasis. However, this method is more challenging and costly (Hoffman, 2015; Stewart et al., 2017). On the other hand, mouse strains used for PDX model construction are also being continuously improved. Severe immunodeficient mice lacking B cells or NK cells, such as SCID, NOD-SCID, and NSG mice, have further increased the success rate of PDX models (Shultz et al., 2005; Morton and Houghton, 2007).

Qingyang Gu et al. established 65 PDX models (approximately 26%) using subcutaneous transplantation in SCID mice from tumor tissues of 254 HCC patients (Gu et al., 2015). The histological morphology and differentiation degree of the models were highly similar to the original tumors, and they retained the intratumoral heterogeneity of the original tumors. In a recent study, it was found that HCC tissues obtained from needle biopsies can also be used to construct PDX models. In this study, 11 PDX models were successfully generated from HCC tissues obtained from needle biopsies of 54 HCC patients, and the PDX models maintained the histological, transcriptomic, and genomic characteristics of the original tumors (Blumer et al., 2019). However, the success rate of PDX model construction in this study was only 20%, which may be attributed to the limited quantity of tumor tissues. On the other hand, Long Yang et al. established Mini-PDX models by transplanting tumor cells derived from HCC patients into immunodeficient mice (Yang et al., 2021). The Mini-PDX models reduced the time required for tumor formation, and the application of Mini-PDX models after partial hepatectomy guided anti-tumor treatment selection, leading to effective extension of the survival of HCC patients. However, Mini-PDX models cannot simulate the tumor microenvironment and the generation of tumor vasculature, resulting in lower sensitivity to targeted drugs compared to cytotoxic drugs. Sorafenib and lenvatinib, as FDA-approved systemic therapies for HCC, showed similar treatment responses in HCC PDX models compared to the primary patients, indicating that PDX models are effective tools for predicting the response to targeted therapy (Tiao et al., 2016; Gao et al., 2021). In a retrospective analysis, comparing the treatment outcomes of 92 patients with advanced solid tumors with the sensitivity of the corresponding PDX models to the same drugs, the sensitivity and specificity of PDX drug screening were 96% and 70%, respectively, with a positive predictive value of 85% and a negative predictive value of 91% (Izumchenko et al., 2017).

Traditional liver cancer PDX models have low immune function and are not suitable for simulating immune responses in the tumor microenvironment (Shultz et al., 2007). To study liver cancer immunotherapy, it is crucial to replicate the complexity of the human immune system. One approach is to use humanized mouse models with a human immune system. The most common method to create humanized mouse models is by transplanting human hematopoietic stem cells and progenitor cells into mouse bone marrow to facilitate immune system development (Rongvaux et al., 2014). Zhao et al. transplanted hematopoietic stem cells into NSG mice to reconstruct a humanized immune system that matches human leukocyte antigens, thus creating a dual-humanized PDX model. The study showed that the engrafted tumors can suppress immune responses and evade immune regulation by altering gene expression profiles, which is a major difference compared to traditional PDX models and provides a new option for investigating the interaction between tumors and the immune system. Humanized PDX models demonstrated a more pronounced growth inhibition in response to immune checkpoint inhibitors compared to NSG mouse PDX models (Zhao et al., 2018). Ideally, hematopoietic stem cells and tumor tissues from liver cancer patients should be transplanted into the same immunodeficient mice, but this approach is cost-prohibitive and technically challenging. Addressing this issue would be a significant step towards developing more personalized PDX models.

In summary, PDX models have unique advantages in studying the mechanisms of liver cancer, identifying biomarkers, and screening drugs. However, limitations should be considered in their application (Zanella et al., 2022). Similar to patient-derived organoids, tumor tissues used to construct PDX models have anatomical bias due to tumor heterogeneity, and only a fraction of implanted tumor cells survive. Additionally, human stromal cells in PDX models are rapidly replaced by mouse stromal cells. Exploring gene expression data from liver cancer patients using PDX models requires the use of bioinformatics methods to subtract the gene expression data from the model mice (Wang et al., 2018).

## 3 Summary and outlook

The transition from 2D cell culture to 3D *in vitro* models has provided a more reliable platform for studying the molecular mechanisms, drug development, and personalized treatment of liver cancer. Emerging technologies such as 3D bioprinting and microfluidics have facilitated the development of 3D culture of liver cancer cells *in vitro*, while hydrogel technologies provide a basic support for 3D culture, allowing the constructed liver cancer models to better mimic the *in vivo* tumor and its microenvironment. Organoids derived from PLC patient tumor tissues and PDX models preserve the genetic characteristics and histological morphology of the primary tumors to the greatest extent, which is of great significance for exploring the mechanisms of liver cancer development and precision oncology. In conclusion, the rapid development of 3D tumor models provides new options for further understanding and treating PLC.

However, the current techniques for constructing PLC models cannot fully replicate the complexity of tumors *in vivo*, and we need to evaluate the advantages and limitations of each model in their application. 3D cell culture techniques, including 3D bioprinting and microfluidic organ-on-a-chip systems, are more efficient in studying cell-cell interactions and high-throughput drug screening, and they also reduce the burden of animal models such as mice. Organoids and PDX models have unique advantages in exploring the development of PLC and personalized tumor treatment, but they are time-consuming and have a relatively low success rate. Studies have shown that co-culturing liver organoids with endothelial cells to generate vascularized organoids significantly improves liver cell-specific functions, indicating the value of vascularization in the generation of liver cancer organoids and PDX models (Yu, 2021). Additionally, the combination of organoids with microfluidic and 3D printing technologies can enhance the biomimetic and complex nature of the 3D models, and significant potential has been explored in the field of organ-on-a-chip systems (Hiratsuka et al., 2022).

As the value of immunotherapy in the treatment of PLC continues to emerge, future efforts should focus on optimizing and using organoids co-cultured with patient-derived tumor tissues and immune cells, as well as developing more standardized humanized PDX models.

## References

[B1] Ahn J. Sei Y.J. Jeon N.L. Kim Y. (2017). Tumor microenvironment on a chip: the progress and future perspective. Bioeng. (Basel) 4 (3), 64. 10.3390/bioengineering4030064 PMC5615310 28952543

[B2] Al Hrout A. Cervantes-Gracia K. Chahwan R. Amin A. (2022). Modelling liver cancer microenvironment using a novel 3D culture system. Sci. Rep. 12 (1), 8003. 10.1038/s41598-022-11641-7 35568708 PMC9107483

[B3] Anderson N.M. Simon M.C. (2020). The tumor microenvironment. Curr. Biol. 30 (16), R921–r925. 10.1016/j.cub.2020.06.081 32810447 PMC8194051

[B4] Ao Z. Cai H. Wu Z. Hu L. Li X. Kaurich C. (2022). Evaluation of cancer immunotherapy using mini-tumor chips. Theranostics 12 (8), 3628–3636. 10.7150/thno.71761 35664082 PMC9131272

[B5] Artegiani B. van Voorthuijsen L. Lindeboom R.G.H. Seinstra D. Heo I. Tapia P. (2019). Probing the tumor suppressor function of BAP1 in CRISPR-engineered human liver organoids. Cell Stem Cell 24 (6), 927–943.e6. 10.1016/j.stem.2019.04.017 31130514

[B6] Badr-Eldin S.M. Aldawsari H.M. Kotta S. Deb P.K. Venugopala K.N. (2022). Three-dimensional *in vitro* cell culture models for efficient drug discovery: progress so far and future prospects. Pharmaceuticals 15 (8), 926. 10.3390/ph15080926 36015074 PMC9412659

[B7] Bershteyn M. Nowakowski T.J. Pollen A.A. Di Lullo E. Nene A. Wynshaw-Boris A. (2017). Human iPSC-derived cerebral organoids model cellular features of lissencephaly and reveal prolonged mitosis of outer radial glia. Cell Stem Cell 20 (4), 435–449.e4. 10.1016/j.stem.2016.12.007 28111201 PMC5667944

[B8] Bertassoni L.E. Cecconi M. Manoharan V. Nikkhah M. Hjortnaes J. Cristino A.L. (2014). Hydrogel bioprinted microchannel networks for vascularization of tissue engineering constructs. Lab. Chip 14 (13), 2202–2211. 10.1039/c4lc00030g 24860845 PMC4201051

[B9] Bhattacharya M. Malinen M.M. Lauren P. Lou Y.R. Kuisma S.W. Kanninen L. (2012). Nanofibrillar cellulose hydrogel promotes three-dimensional liver cell culture. J. Control Release 164 (3), 291–298. 10.1016/j.jconrel.2012.06.039 22776290

[B10] Bhise N.S. Manoharan V. Massa S. Tamayol A. Ghaderi M. Miscuglio M. (2016). A liver-on-a-chip platform with bioprinted hepatic spheroids. Biofabrication 8 (1), 014101. 10.1088/1758-5090/8/1/014101 26756674

[B11] Blumer T. Fofana I. Matter M.S. Wang X. Montazeri H. Calabrese D. (2019). Hepatocellular carcinoma xenografts established from needle biopsies preserve the characteristics of the originating tumors. Hepatol. Commun. 3 (7), 971–986. 10.1002/hep4.1365 31334445 PMC6601318

[B12] Brancato V. Oliveira J.M. Correlo V.M. Reis R.L. Kundu S.C. (2020). Could 3D models of cancer enhance drug screening? Biomaterials 232, 119744. 10.1016/j.biomaterials.2019.119744 31918229

[B13] Bresnahan E. Ramadori P. Heikenwalder M. Zender L. Lujambio A. (2020). Novel patient-derived preclinical models of liver cancer. J. Hepatol. 72 (2), 239–249. 10.1016/j.jhep.2019.09.028 31954489

[B14] Broutier L. Andersson-Rolf A. Hindley C.J. Boj S.F. Clevers H. Koo B.K. (2016). Culture and establishment of self-renewing human and mouse adult liver and pancreas 3D organoids and their genetic manipulation. Nat. Protoc. 11 (9), 1724–1743. 10.1038/nprot.2016.097 27560176

[B15] Broutier L. Mastrogiovanni G. Verstegen M.M. Francies H.E. Gavarró L.M. Bradshaw C.R. (2017). Human primary liver cancer-derived organoid cultures for disease modeling and drug screening. Nat. Med. 23 (12), 1424–1435. 10.1038/nm.4438 29131160 PMC5722201

[B16] Brown Z.J. Heinrich B. Greten T.F. (2018). Mouse models of hepatocellular carcinoma: an overview and highlights for immunotherapy research. Nat. Rev. Gastroenterol. Hepatol. 15 (9), 536–554. 10.1038/s41575-018-0033-6 29904153

[B17] Caleffi J.T. Aal M.C.E. Gallindo H.O.M. Caxali G.H. Crulhas B.P. Ribeiro A.O. (2021). Magnetic 3D cell culture: state of the art and current advances. Life Sci. 286, 120028. 10.1016/j.lfs.2021.120028 34627776

[B18] Calitz C. Pavlović N. Rosenquist J. Zagami C. Samanta A. Heindryckx F. (2020). A biomimetic model for liver cancer to study tumor-stroma interactions in a 3D environment with tunable bio-physical properties. J. Vis. Exp. 162. 10.3791/61606 32831309

[B19] Decante G. Costa J.B. Silva-Correia J. Collins M.N. Reis R.L. Oliveira J.M. (2021). Engineering bioinks for 3D bioprinting. Biofabrication 13 (3), 032001. 10.1088/1758-5090/abec2c 33662949

[B20] De Crignis E. Hossain T. Romal S. Carofiglio F. Moulos P. Khalid M.M. (2021). Application of human liver organoids as a patient-derived primary model for HBV infection and related hepatocellular carcinoma. Elife 10, e60747. 10.7554/eLife.60747 34328417 PMC8384419

[B21] de Lau W. Barker N. Low T.Y. Koo B.K. Li V.S. Teunissen H. (2011). Lgr5 homologues associate with Wnt receptors and mediate R-spondin signalling. Nature 476 (7360), 293–297. 10.1038/nature10337 21727895

[B22] De Minicis S. Kisseleva T. Francis H. Baroni G.S. Benedetti A. Brenner D. (2013). Liver carcinogenesis: rodent models of hepatocarcinoma and cholangiocarcinoma. Dig. Liver Dis. 45 (6), 450–459. 10.1016/j.dld.2012.10.008 23177172 PMC3716909

[B23] De Santis M.M. Alsafadi H.N. Tas S. Bölükbas D.A. Prithiviraj S. Da Silva I.A.N. (2021). Extracellular-matrix-reinforced bioinks for 3D bioprinting human tissue. Adv. Mater 33 (3), e2005476. 10.1002/adma.202005476 33300242 PMC11469085

[B24] Dijkstra K.K. Cattaneo C.M. Weeber F. Chalabi M. van de Haar J. Fanchi L.F. (2018). Generation of tumor-reactive T cells by Co-culture of peripheral blood lymphocytes and tumor organoids. Cell 174 (6), 1586–1598.e12. 10.1016/j.cell.2018.07.009 30100188 PMC6558289

[B25] Dong R. Zhang B. Zhang X. (2022). Liver organoids: an *in vitro* 3D model for liver cancer study. Cell Biosci. 12 (1), 152. 10.1186/s13578-022-00890-8 36085085 PMC9463833

[B26] Drury J.L. Mooney D.J. (2003). Hydrogels for tissue engineering: scaffold design variables and applications. Biomaterials 24 (24), 4337–4351. 10.1016/s0142-9612(03)00340-5 12922147

[B27] Forner A. Reig M. Bruix J. (2018). Hepatocellular carcinoma. Lancet 391 (10127), 1301–1314. 10.1016/s0140-6736(18)30010-2 29307467

[B28] Franchi-Mendes T. Lopes N. Brito C. (2021). Heterotypic tumor spheroids in agitation-based cultures: a scaffold-free cell model that sustains long-term survival of endothelial cells. Front. Bioeng. Biotechnol. 9, 649949. 10.3389/fbioe.2021.649949 34178955 PMC8219978

[B29] Gao H. Korn J.M. Ferretti S. Monahan J.E. Wang Y. Singh M. (2015). High-throughput screening using patient-derived tumor xenografts to predict clinical trial drug response. Nat. Med. 21 (11), 1318–1325. 10.1038/nm.3954 26479923

[B30] Gao Y. Zhou R. Huang J.F. Hu B. Cheng J.W. Huang X.W. (2021). Patient-derived xenograft models for intrahepatic cholangiocarcinoma and their application in guiding personalized medicine. Front. Oncol. 11, 704042. 10.3389/fonc.2021.704042 34327143 PMC8315044

[B31] Greene T. Lin C.C. (2015). Modular cross-linking of gelatin-based thiol-norbornene hydrogels for *in vitro* 3D culture of hepatocellular carcinoma cells. ACS Biomater. Sci. Eng. 1 (12), 1314–1323. 10.1021/acsbiomaterials.5b00436 33429678

[B32] Gu Q. Zhang B. Sun H. Xu Q. Tan Y. Wang G. (2015). Genomic characterization of a large panel of patient-derived hepatocellular carcinoma xenograft tumor models for preclinical development. Oncotarget 6 (24), 20160–20176. 10.18632/oncotarget.3969 26062443 PMC4652995

[B33] Hiratsuka K. Miyoshi T. Kroll K.T. Gupta N.R. Valerius M.T. Ferrante T. (2022). Organoid-on-a-chip model of human ARPKD reveals mechanosensing pathomechanisms for drug discovery. Sci. Adv. 8 (38), eabq0866. 10.1126/sciadv.abq0866 36129975 PMC9491724

[B34] Hoffman R.M. (2015). Patient-derived orthotopic xenografts: better mimic of metastasis than subcutaneous xenografts. Nat. Rev. Cancer 15 (8), 451–452. 10.1038/nrc3972 26422835

[B35] Huang Y. Zhang X.F. Gao G. Yonezawa T. Cui X. (2017). 3D bioprinting and the current applications in tissue engineering. Biotechnol. J. 12 (8). 10.1002/biot.201600734 28675678

[B36] Izumchenko E. Paz K. Ciznadija D. Sloma I. Katz A. Vasquez-Dunddel D. (2017). Patient-derived xenografts effectively capture responses to oncology therapy in a heterogeneous cohort of patients with solid tumors. Ann. Oncol. 28 (10), 2595–2605. 10.1093/annonc/mdx416 28945830 PMC5834154

[B37] Ji S. Feng L. Fu Z. Wu G. Wu Y. Lin Y. (2023). Pharmaco-proteogenomic characterization of liver cancer organoids for precision oncology. Sci. Transl. Med. 15 (706), eadg3358. 10.1126/scitranslmed.adg3358 37494474 PMC10949980

[B38] Jia D. Liu C. Zhu Z. Cao Y. Wen W. Hong Z. (2022). Novel transketolase inhibitor oroxylin A suppresses the non-oxidative pentose phosphate pathway and hepatocellular carcinoma tumour growth in mice and patient-derived organoids. Clin. Transl. Med. 12 (11), e1095. 10.1002/ctm2.1095 36314067 PMC9619225

[B39] Kim B.S. Das S. Jang J. Cho D.W. (2020). Decellularized extracellular matrix-based bioinks for engineering tissue- and organ-specific microenvironments. Chem. Rev. 120 (19), 10608–10661. 10.1021/acs.chemrev.9b00808 32786425

[B40] Kim M.K. Jeong W. Jeon S. Kang H.W. (2023). 3D bioprinting of dECM-incorporated hepatocyte spheroid for simultaneous promotion of cell-cell and -ECM interactions. Front. Bioeng. Biotechnol. 11, 1305023. 10.3389/fbioe.2023.1305023 38026892 PMC10679743

[B41] Kronemberger G.S. Miranda G. Tavares R.S.N. Montenegro B. Kopke Ú A. Baptista L.S. (2021). Recapitulating tumorigenesis *in vitro*: opportunities and challenges of 3D bioprinting. Front. Bioeng. Biotechnol. 9, 682498. 10.3389/fbioe.2021.682498 34239860 PMC8258101

[B42] Kudo M. Finn R.S. Qin S. Han K.H. Ikeda K. Piscaglia F. (2018). Lenvatinib versus sorafenib in first-line treatment of patients with unresectable hepatocellular carcinoma: a randomised phase 3 non-inferiority trial. Lancet 391 (10126), 1163–1173. 10.1016/s0140-6736(18)30207-1 29433850

[B43] Kuo W.S. Chang C.Y. Chuang H.Y. Su P.L. Wang J.Y. Wu P.C. (2023). Corrigendum to "Single-sized N-functionality graphene quantum dot in tunable dual-modality near infrared-I/II illumination detection and photodynamic therapy under multiphoton nonlinear excitation" [Biosens. Bioelectron. 241 (2023) 115648/10.1016/j.bios.2023.115648]. Biosens. Bioelectron. 2023, 115870. 10.1016/j.bios.2023.115870 37690354

[B44] Lam Y.K. Yu J. Huang H. Ding X. Wong A.M. Leung H.H. (2023). TP53 R249S mutation in hepatic organoids captures the predisposing cancer risk. Hepatology 78 (3), 727–740. 10.1002/hep.32802 36221953 PMC10086078

[B45] Lee J. Lee S. Kim S.M. Shin H. (2021). Size-controlled human adipose-derived stem cell spheroids hybridized with single-segmented nanofibers and their effect on viability and stem cell differentiation. Biomater. Res. 25 (1), 14. 10.1186/s40824-021-00215-9 33902733 PMC8074457

[B46] Lee J.W. Choi Y.J. Yong W.J. Pati F. Shim J.H. Kang K.S. (2016). Development of a 3D cell printed construct considering angiogenesis for liver tissue engineering. Biofabrication 8 (1), 015007. 10.1088/1758-5090/8/1/015007 26756962

[B47] Lee S.W.L. Adriani G. Ceccarello E. Pavesi A. Tan A.T. Bertoletti A. (2018). Characterizing the role of monocytes in T cell cancer immunotherapy using a 3D microfluidic model. Front. Immunol. 9, 416. 10.3389/fimmu.2018.00416 29559973 PMC5845585

[B48] Lei M. Wang X. (2016). Biodegradable polymers and stem cells for bioprinting. Molecules 21 (5), 539. 10.3390/molecules21050539 27136526 PMC6274354

[B49] Leung M. Kievit F.M. Florczyk S.J. Veiseh O. Wu J. Park J.O. (2010). Chitosan-alginate scaffold culture system for hepatocellular carcinoma increases malignancy and drug resistance. Pharm. Res. 27 (9), 1939–1948. 10.1007/s11095-010-0198-3 20585843 PMC2917619

[B50] Li C. Jin B. Sun H. Wang Y. Zhao H. Sang X. (2022). Exploring the function of stromal cells in cholangiocarcinoma by three-dimensional bioprinting immune microenvironment model. Front. Immunol. 13, 941289. 10.3389/fimmu.2022.941289 35983036 PMC9378822

[B51] Li L. Knutsdottir H. Hui K. Weiss M.J. He J. Philosophe B. (2019a). Human primary liver cancer organoids reveal intratumor and interpatient drug response heterogeneity. JCI Insight 4 (2), e121490. 10.1172/jci.insight.121490 30674722 PMC6413833

[B52] Li Y. Zhang T. Pang Y. Li L. Chen Z.N. Sun W. (2019b). 3D bioprinting of hepatoma cells and application with microfluidics for pharmacodynamic test of Metuzumab. Biofabrication 11 (3), 034102. 10.1088/1758-5090/ab256c 31141796

[B53] Liaw C.Y. Ji S. Guvendiren M. (2018). Engineering 3D hydrogels for personalized *in vitro* human tissue models. Adv. Healthc. Mater 7 (4). 10.1002/adhm.201701165 29345429

[B54] Lim J.T.C. Kwang L.G. Ho N.C.W. Toh C.C.M. Too N.S.H. Hooi L. (2022). Hepatocellular carcinoma organoid co-cultures mimic angiocrine crosstalk to generate inflammatory tumor microenvironment. Biomaterials 284, 121527. 10.1016/j.biomaterials.2022.121527 35483200

[B55] Liu C. Qin T. Huang Y. Li Y. Chen G. Sun C. (2020). Drug screening model meets cancer organoid technology. Transl. Oncol. 13 (11), 100840. 10.1016/j.tranon.2020.100840 32822897 PMC7451679

[B56] Llovet J.M. Castet F. Heikenwalder M. Maini M.K. Mazzaferro V. Pinato D.J. (2022). Immunotherapies for hepatocellular carcinoma. Nat. Rev. Clin. Oncol. 19 (3), 151–172. 10.1038/s41571-021-00573-2 34764464

[B57] Lu S. Cuzzucoli F. Jiang J. Liang L.G. Wang Y. Kong M. (2018). Development of a biomimetic liver tumor-on-a-chip model based on decellularized liver matrix for toxicity testing. Lab. Chip 18 (22), 3379–3392. 10.1039/c8lc00852c 30298144

[B58] Mao S. He J. Zhao Y. Liu T. Xie F. Yang H. (2020). Bioprinting of patient-derived *in vitro* intrahepatic cholangiocarcinoma tumor model: establishment, evaluation and anti-cancer drug testing. Biofabrication 12 (4), 045014. 10.1088/1758-5090/aba0c3 32599574

[B59] Meng F. Meyer C.M. Joung D. Vallera D.A. McAlpine M.C. Panoskaltsis-Mortari A. (2019). 3D bioprinted *in vitro* metastatic models via reconstruction of tumor microenvironments. Adv. Mater 31 (10), e1806899. 10.1002/adma.201806899 30663123 PMC6996245

[B60] Morton C.L. Houghton P.J. (2007). Establishment of human tumor xenografts in immunodeficient mice. Nat. Protoc. 2 (2), 247–250. 10.1038/nprot.2007.25 17406581

[B61] Moscato S. Ronca F. Campani D. Danti S. (2015). Poly(vinyl alcohol)/gelatin hydrogels cultured with HepG2 cells as a 3D model of hepatocellular carcinoma: a morphological study. J. Funct. Biomater. 6 (1), 16–32. 10.3390/jfb6010016 25590431 PMC4384098

[B62] Naruse M. Masui R. Ochiai M. Maru Y. Hippo Y. Imai T. (2020). An organoid-based carcinogenesis model induced by *in vitro* chemical treatment. Carcinogenesis 41 (10), 1444–1453. 10.1093/carcin/bgaa011 32047892

[B63] Nguyen R. Da Won Bae S. Qiao L. George J. (2021). Developing liver organoids from induced pluripotent stem cells (iPSCs): an alternative source of organoid generation for liver cancer research. Cancer Lett. 508, 13–17. 10.1016/j.canlet.2021.03.017 33771683

[B64] Nikolova M.P. Chavali M.S. (2019). Recent advances in biomaterials for 3D scaffolds: a review. Bioact. Mater 4, 271–292. 10.1016/j.bioactmat.2019.10.005 31709311 PMC6829098

[B65] Nuciforo S. Fofana I. Matter M.S. Blumer T. Calabrese D. Boldanova T. (2018). Organoid models of human liver cancers derived from tumor needle biopsies. Cell Rep. 24 (5), 1363–1376. 10.1016/j.celrep.2018.07.001 30067989 PMC6088153

[B66] Ouchi R. Togo S. Kimura M. Shinozawa T. Koido M. Koike H. (2019). Modeling steatohepatitis in humans with pluripotent stem cell-derived organoids. Cell Metab. 30 (2), 374–384.e6. 10.1016/j.cmet.2019.05.007 31155493 PMC6687537

[B67] Ouyang L. Yao R. Mao S. Chen X. Na J. Sun W. (2015). Three-dimensional bioprinting of embryonic stem cells directs highly uniform embryoid body formation. Biofabrication 7 (4), 044101. 10.1088/1758-5090/7/4/044101 26531008

[B68] Peng W.C. Logan C.Y. Fish M. Anbarchian T. Aguisanda F. Álvarez-Varela A. (2018). Inflammatory cytokine TNFα promotes the long-term expansion of primary hepatocytes in 3D culture. Cell 175 (6), 1607–1619.e15. 10.1016/j.cell.2018.11.012 30500539 PMC6497386

[B69] Rongvaux A. Willinger T. Martinek J. Strowig T. Gearty S.V. Teichmann L.L. (2014). Development and function of human innate immune cells in a humanized mouse model. Nat. Biotechnol. 32 (4), 364–372. 10.1038/nbt.2858 24633240 PMC4017589

[B70] Rumgay H. Arnold M. Ferlay J. Lesi O. Cabasag C.J. Vignat J. (2022). Global burden of primary liver cancer in 2020 and predictions to 2040. J. Hepatol. 77 (6), 1598–1606. 10.1016/j.jhep.2022.08.021 36208844 PMC9670241

[B71] Sato K. Zhang W. Safarikia S. Isidan A. Chen A.M. Li P. (2021). Organoids and spheroids as models for studying cholestatic liver injury and cholangiocarcinoma. Hepatology 74 (1), 491–502. 10.1002/hep.31653 33222247 PMC8529583

[B72] Schwank G. Koo B.K. Sasselli V. Dekkers J.F. Heo I. Demircan T. (2013). Functional repair of CFTR by CRISPR/Cas9 in intestinal stem cell organoids of cystic fibrosis patients. Cell Stem Cell 13 (6), 653–658. 10.1016/j.stem.2013.11.002 24315439

[B73] Shah U.K. Mallia J.O. Singh N. Chapman K.E. Doak S.H. Jenkins G.J.S. (2018). Reprint of: a three-dimensional *in vitro* HepG2 cells liver spheroid model for genotoxicity studies. Mutat. Res. Genet. Toxicol. Environ. Mutagen 834, 35–41. 10.1016/j.mrgentox.2018.06.020 30173862

[B74] Shamir E.R. Ewald A.J. (2014). Three-dimensional organotypic culture: experimental models of mammalian biology and disease. Nat. Rev. Mol. Cell Biol. 15 (10), 647–664. 10.1038/nrm3873 25237826 PMC4352326

[B75] Sharma S.V. Haber D.A. Settleman J. (2010). Cell line-based platforms to evaluate the therapeutic efficacy of candidate anticancer agents. Nat. Rev. Cancer 10 (4), 241–253. 10.1038/nrc2820 20300105

[B76] Shinozawa T. Kimura M. Cai Y. Saiki N. Yoneyama Y. Ouchi R. (2021). High-fidelity drug-induced liver injury screen using human pluripotent stem cell-derived organoids. Gastroenterology 160 (3), 831–846.e10. 10.1053/j.gastro.2020.10.002 33039464 PMC7878295

[B77] Shiota J. Samuelson L.C. Razumilava N. (2021). Hepatobiliary organoids and their applications for studies of liver Health and disease: are we there yet? Hepatology 74 (4), 2251–2263. 10.1002/hep.31772 33638203 PMC9067600

[B78] Shultz L.D. Ishikawa F. Greiner D.L. (2007). Humanized mice in translational biomedical research. Nat. Rev. Immunol. 7 (2), 118–130. 10.1038/nri2017 17259968

[B79] Shultz L.D. Lyons B.L. Burzenski L.M. Gott B. Chen X. Chaleff S. (2005). Human lymphoid and myeloid cell development in NOD/LtSz-scid IL2R gamma null mice engrafted with mobilized human hemopoietic stem cells. J. Immunol. 174 (10), 6477–6489. 10.4049/jimmunol.174.10.6477 15879151

[B80] Stewart E. Federico S.M. Chen X. Shelat A.A. Bradley C. Gordon B. (2017). Orthotopic patient-derived xenografts of paediatric solid tumours. Nature 549 (7670), 96–100. 10.1038/nature23647 28854174 PMC5659286

[B81] Strelez C. Jiang H.Y. Mumenthaler S.M. (2023). Organs-on-chips: a decade of innovation. Trends Biotechnol. 41 (3), 278–280. 10.1016/j.tibtech.2023.01.004 36658006

[B82] Sun L. Wang Y. Cen J. Ma X. Cui L. Qiu Z. (2019). Modelling liver cancer initiation with organoids derived from directly reprogrammed human hepatocytes. Nat. Cell Biol. 21 (8), 1015–1026. 10.1038/s41556-019-0359-5 31332348

[B83] Tabrizian P. Jibara G. Shrager B. Schwartz M. Roayaie S. (2015). Recurrence of hepatocellular cancer after resection: patterns, treatments, and prognosis. Ann. Surg. 261 (5), 947–955. 10.1097/sla.0000000000000710 25010665

[B84] Tang J. Cui J. Chen R. Guo K. Kang X. Li Y. (2011). A three-dimensional cell biology model of human hepatocellular carcinoma *in vitro* . Tumour Biol. 32 (3), 469–479. 10.1007/s13277-010-0140-7 21140254

[B85] Tetsuka H. Shin S.R. (2020). Materials and technical innovations in 3D printing in biomedical applications. J. Mater Chem. B 8 (15), 2930–2950. 10.1039/d0tb00034e 32239017 PMC8092991

[B86] Tiao G. Geller J. Timchenko N.A. (2016). Generation of pediatric liver cancer patient-derived xenograft platforms for pediatric liver cancer: a critical stage in the development of anticancer treatments. Hepatology 64 (4), 1017–1019. 10.1002/hep.28711 27359258

[B87] Turner D.A. Girgin M. Alonso-Crisostomo L. Trivedi V. Baillie-Johnson P. Glodowski C.R. (2017). Anteroposterior polarity and elongation in the absence of extra-embryonic tissues and of spatially localised signalling in gastruloids: mammalian embryonic organoids. Development 144 (21), 3894–3906. 10.1242/dev.150391 28951435 PMC5702072

[B88] Tuveson D. Clevers H. (2019). Cancer modeling meets human organoid technology. Science 364 (6444), 952–955. 10.1126/science.aaw6985 31171691

[B89] van Duinen V. Trietsch S.J. Joore J. Vulto P. Hankemeier T. (2015). Microfluidic 3D cell culture: from tools to tissue models. Curr. Opin. Biotechnol. 35, 118–126. 10.1016/j.copbio.2015.05.002 26094109

[B90] Wang T. Lu R. Kapur P. Jaiswal B.S. Hannan R. Zhang Z. (2018). An empirical approach leveraging tumorgrafts to dissect the tumor microenvironment in renal cell carcinoma identifies missing link to prognostic inflammatory factors. Cancer Discov. 8 (9), 1142–1155. 10.1158/2159-8290.Cd-17-1246 29884728 PMC6125163

[B91] Wang W. Yuan T. Ma L. Zhu Y. Bao J. Zhao X. (2022). Hepatobiliary tumor organoids reveal HLA class I neoantigen landscape and antitumoral activity of neoantigen peptide enhanced with immune checkpoint inhibitors. Adv. Sci. (Weinh) 9 (22), e2105810. 10.1002/advs.202105810 35665491 PMC9353440

[B92] Wang Y. Kankala R.K. Zhang J. Hao L. Zhu K. Wang S. (2020). Modeling endothelialized hepatic tumor microtissues for drug screening. Adv. Sci. (Weinh) 7 (21), 2002002. 10.1002/advs.202002002 33173735 PMC7610277

[B93] Xian L. Zhao P. Chen X. Wei Z. Ji H. Zhao J. (2022). Heterogeneity, inherent and acquired drug resistance in patient-derived organoid models of primary liver cancer. Cell Oncol. (Dordr) 45 (5), 1019–1036. 10.1007/s13402-022-00707-3 36036881 PMC12978112

[B94] Xiang Y. Miller K. Guan J. Kiratitanaporn W. Tang M. Chen S. (2022). 3D bioprinting of complex tissues *in vitro*: state-of-the-art and future perspectives. Arch. Toxicol. 96 (3), 691–710. 10.1007/s00204-021-03212-y 35006284 PMC8850226

[B95] Xie F. Sun L. Pang Y. Xu G. Jin B. Xu H. (2021). Three-dimensional bio-printing of primary human hepatocellular carcinoma for personalized medicine. Biomaterials 265, 120416. 10.1016/j.biomaterials.2020.120416 33007612

[B96] Xu S. Ling S. Shan Q. Ye Q. Zhan Q. Jiang G. (2021). Self-activated cascade-responsive sorafenib and USP22 shRNA Co-delivery system for synergetic hepatocellular carcinoma therapy. Adv. Sci. (Weinh) 8 (5), 2003042. 10.1002/advs.202003042 33717848 PMC7927615

[B97] Yang L. Yuan Z. Zhang Y. Cui Z. Li Y. Hou J. (2021). MiniPDX-guided postoperative anticancer treatment can effectively prolong the survival of patients with hepatocellular carcinoma. Cancer Chemother. Pharmacol. 87 (1), 125–134. 10.1007/s00280-020-04182-1 33141330

[B98] Yi H.G. Lee H. Cho D.W. (2017). 3D printing of organs-on-chips. Bioeng. (Basel) 4 (1), 10. 10.3390/bioengineering4010010 PMC5590440 28952489

[B99] Yip D. Cho C.H. (2013). A multicellular 3D heterospheroid model of liver tumor and stromal cells in collagen gel for anti-cancer drug testing. Biochem. Biophys. Res. Commun. 433 (3), 327–332. 10.1016/j.bbrc.2013.03.008 23501105

[B100] Yu J. (2021). Vascularized organoids: a more complete model. Int. J. Stem Cells 14 (2), 127–137. 10.15283/ijsc20143 33377457 PMC8138664

[B101] Yun C. Kim S.H. Jung Y.S. (2022). Current research trends in the application of *in vitro* three-dimensional models of liver cells. Pharmaceutics 15 (1), 54. 10.3390/pharmaceutics15010054 36678683 PMC9866911

[B102] Zanella E.R. Grassi E. Trusolino L. (2022). Towards precision oncology with patient-derived xenografts. Nat. Rev. Clin. Oncol. 19 (11), 719–732. 10.1038/s41571-022-00682-6 36151307

[B103] Zhang D. Li L. Jiang H. Li Q. Wang-Gillam A. Yu J. (2018). Tumor-stroma il1β-IRAK4 feedforward circuitry drives tumor fibrosis, chemoresistance, and poor prognosis in pancreatic cancer. Cancer Res. 78 (7), 1700–1712. 10.1158/0008-5472.Can-17-1366 29363544 PMC5890818

[B104] Zhao Y. Li Z.X. Zhu Y.J. Fu J. Zhao X.F. Zhang Y.N. (2021). Single-cell transcriptome analysis uncovers intratumoral heterogeneity and underlying mechanisms for drug resistance in hepatobiliary tumor organoids. Adv. Sci. (Weinh) 8 (11), e2003897. 10.1002/advs.202003897 34105295 PMC8188185

[B105] Zhao Y. Shuen T.W.H. Toh T.B. Chan X.Y. Liu M. Tan S.Y. (2018). Development of a new patient-derived xenograft humanised mouse model to study human-specific tumour microenvironment and immunotherapy. Gut 67 (10), 1845–1854. 10.1136/gutjnl-2017-315201 29602780 PMC6145285

[B106] Zhuang X. Deng G. Wu X. Xie J. Li D. Peng S. (2023). Recent advances of three-dimensional bioprinting technology in hepato-pancreato-biliary cancer models. Front. Oncol. 13, 1143600. 10.3389/fonc.2023.1143600 37188191 PMC10175665

[B107] Zuchowska A. Kwapiszewska K. Chudy M. Dybko A. Brzozka Z. (2017). Studies of anticancer drug cytotoxicity based on long-term HepG2 spheroid culture in a microfluidic system. Electrophoresis 38 (8), 1206–1216. 10.1002/elps.201600417 28090668

